# Comparison of prognostic factors between bacteraemic and non-bacteraemic critically ill immunocompetent patients in community-acquired severe pneumococcal pneumonia: a STREPTOGENE sub-study

**DOI:** 10.1186/s13613-021-00936-z

**Published:** 2021-10-24

**Authors:** Hugo Bellut, Raphael Porcher, Emmanuelle Varon, Pierre Asfar, Yves Le Tulzo, Bruno Megarbane, Armelle Mathonnet, Anthony Dugard, Anne Veinstein, Kader Ouchenir, Shidasp Siami, Jean Reignier, Arnaud Galbois, Joël Cousson, Sébastien Preau, Olivier Baldesi, Jean‑Philippe Rigaud, Bertrand Souweine, Benoit Misset, Frederic Jacobs, Florent Dewavrin, Jean‑Paul Mira, Jean‑Pierre Bedos

**Affiliations:** 1 Réanimation Médico‑Chirurgicale, Hôpital A. Mignot, CH Versailles, 177 Rue de Versailles, 78157 Le Chesnay, France; 2 Centre de Recherche Épidémiologie et Statistique Sorbonne Paris Cité (CRESS‑UMR1153), Inserm, Centre d’épidémiologie clinique, Centre Equator France, Hôpital Hôtel-Dieu, Université Paris Descartes, 75004 Paris, France; 3 Laboratoire de Microbiologie, Centre National de Référence des Pneumocoques, AP-HP Hôpital Européen Georges-Pompidou, 75908 Paris Cedex 15, France; 4 Centre National de Référence des Pneumocoques, Centre Hospitalier Interrcommunal de Créteil, 94000 Créteil, France; 5 Réanimation Médicale, CHU Angers, 49933 Angers Cedex 9, France; 6 CHU Rennes, SMIRM, 35033 Rennes Cedex 9, France; 7 Réanimation Médicale et Toxicologique, Hôpital Lariboisière, 75010 Paris, France; 8 Réanimation Polyvalente, Hôpital de La Source, 45067 Orléans Cedex 2, France; 9 Réanimation Polyvalente, CHU Dupuytren, 87042 Limoges, France; 10 Réanimation, CHU Jean Bernard, 86021 Poitiers Cedex, France; 11 Réanimation, Hôpital Louis Pasteur, 28018 Chartres Cedex, France; 12 Réanimation Polyvalente, CH Sud Essonne, 91152 Etampes Cedex 02, France; 13 Réanimation Médicale, CHU Nantes, 44093 Nantes Cedex 1, France; 14 Réanimation Médicale, Hôpital St Antoine, 75012 Paris, France; 15 Réanimation Polyvalente, Hôpital Robert Debré, 51092 Reims Cedex, France; 16 Réanimation, Hôpital A. Calmette, 59037 Lille Cedex, France; 17 Réanimation, CH du Pays d’Aix, 13616 Aix En Provence, France; 18 Réanimation Polyvalente, CH Dieppe, 76202 Dieppe Cedex, France; 19 Réanimation Médicale, CHU Gabriel Montpied, 63000 Clermont Ferrand, France; 20 Réanimation, Hôpital Saint Joseph, 75014 Paris, France; 21 Réanimation Médicale, Hôpital Antoine Béclère, 92140 Clamart, France; 22 Réanimation, CH Valenciennes, 59300 Valenciennes, France; 23 Réanimation Médicale, Hôpital Cochin, 75679 Paris Cedex 14, France

**Keywords:** Pneumococcal pneumonia, Pneumococcal bacteraemia, Severe community-acquired pneumonia, Macrolides, Fluoroquinolones

## Abstract

**Background:**

The presence of bacteraemia in pneumococcal pneumonia in critically ill patients does not appear to be a strong independent prognostic factor in the existing literature. However, there may be a specific pattern of factors associated with mortality for ICU patients with bacteraemic pneumococcal community-acquired pneumonia (CAP). We aimed to compare the factors associated with mortality, according to the presence of bacteraemia or not on admission, for patients hospitalised in intensive care for severe pneumococcal CAP.

**Methods:**

This was a post hoc analysis of data from the prospective, observational, multicentre STREPTOGENE study in immunocompetent Caucasian adults admitted to intensive care in France between 2008 and 2012 for pneumococcal CAP. Patients were divided into two groups based on initial blood culture (positive vs. negative) for *Streptococcus pneumoniae*. The primary outcome was hospital mortality, which was compared between the two groups using odds ratios according to predefined variables to search for a prognostic interaction present in bacterial patients but not non-bacteraemic patients. Potential differences in the distribution of serotypes between the two groups were assessed. The prognostic consequences of the presence or not of initial bi-antibiotic therapy were assessed, specifically in bacteraemic patients.

**Results:**

Among 614 included patients, 274 had a blood culture positive for *S. pneumoniae* at admission and 340 did not. The baseline difference between the groups was more frequent leukopaenia (26% vs. 14%, *p* = 0.0002) and less frequent pre-hospital antibiotic therapy (10% vs. 16.3%, *p* = 0.024) for the bacteraemic patients. Hospital mortality was not significantly different between the two groups (*p* = 0.11). We did not observe any prognostic factors specific to the bacteraemic patient population, as the statistical comparison of the odds ratios, as an indication of the association between the predefined prognostic parameters and mortality, showed them to be similar for the two groups. Bacteraemic patients more often had invasive serotypes but less often serotypes associated with high case fatality rates (*p* = 0.003). The antibiotic regimens were similar for the two groups. There was no difference in mortality for patients in either group given a beta-lactam alone vs. a beta-lactam combined with a macrolide or fluoroquinolone.

**Conclusion:**

Bacteraemia had no influence on the mortality of immunocompetent Caucasian adults admitted to intensive care for severe pneumococcal CAP, regardless of the profile of the associated prognostic factors.

**Supplementary Information:**

The online version contains supplementary material available at 10.1186/s13613-021-00936-z.

## Background

*Streptococcus pneumoniae* is the main cause of severe bacterial community-acquired pneumonia (CAP), requiring management in the intensive care unit (ICU) [1]. Bacteraemia affects approximately 40% of patients admitted to intensive care for pneumococcal CAP [2–4]. The presence of bacteraemia has long been considered to be a marker of severity [5–9] that can potentially warrant a change in patient management. However, studies on the prognostic significance of bacteraemia have provided conflicting results [3, 10, 11].

Most studies comparing monotherapy (with a beta-lactam or fluoroquinolone) to dual therapy (a beta-lactam and a macrolide or fluoroquinolone) for the treatment of pneumococcal CAP found better outcomes with dual therapy, notably for bacteraemic patients admitted to medical wards [12–15]. However, these studies were generally retrospective and included few patients managed in the ICU [15, 16]. Moreover, the benefits observed with the beta-lactam-macrolide combination were absent for patients enrolled in randomised controlled trials and those given antibiotics according to guidelines [17]. Few studies have investigated the prognostic factors of patients with bacteraemic CAP [5, 7, 18, 19], particularly those admitted to the ICU.

Although bacteraemia does not appear to be a strong independent prognostic factor, there may be a specific pattern of factors associated with mortality in ICU patients with bacteraemic pneumococcal CAP. Furthermore, given the specific vulnerability of ICU patients, a reappraisal of the various treatment modalities and their effects on survival is merited.

The main objective of this study was to compare the prognostic factors between bacteraemic and non-bacteraemic patients in the context of severe pneumococcal CAP. The secondary objectives were to compare the distribution of serotype patterns and to observe the prognostic consequences of receiving or not, initial dual antibiotic therapy according to the presence, or not, of bacteraemia.

## Methods

### Study design

This study consisted of the post hoc analyses of data from the multicentre, prospective, observational study STREPTOGENE [2], which analysed the relative contribution of various factors, including patient characteristics, pneumococcal serotypes, and antibiotic regimens, to the outcome of severe pneumococcal CAP of ICU patients. Consecutive immunocompetent Caucasians > 18 years of age admitted to multiple French ICUs in university- and non-university-affiliated hospitals between 2008 and 2012 for pneumococcal CAP were included.

The STREPTOGENE study sponsor registered the study database with the French Data Protection Authority (Commission Nationale de l’Informatique et des Libertés, ENRCNIL 909234). The study project was approved by the appropriate ethics committee (Comité de Protection des Personnes d’Ile de France, September 9, 2008, #2008/36NICB). Each investigator undertook to conduct the study in compliance with Good Clinical Practice guidelines and the 1964 Declaration of Helsinki and its amendments. Written informed consent was obtained before study inclusion from patients who were competent. For patients who were not competent, written informed consent was obtained from the next-of-kin and then from the patients as soon as they were able. For the present sub-study, the ethics committee (Comité de Protection des Personnes d’Ile de France) confirmed that the study data were anonymised and therefore waived the need for informed consent.

### Inclusion and exclusion criteria

All patients included met the CAP criteria, with acute respiratory manifestations and a new infiltrate by chest radiography. The pneumococcal infection was documented by a positive urinary *S. pneumoniae* antigen test and/or cultures of respiratory specimens (sputum, tracheal aspirate, distal protected airway specimen, pleural aspirate) and/or cultures of blood samples. Severe CAP was defined according to the American Thoracic Society as at least one of two major criteria (invasive mechanical ventilation [IMV] or septic shock) or at least three of the following minor criteria: respiratory rate > 30/min, PaO_2_/FiO_2_ < 250 or non-invasive ventilation (NIV), multilobar infiltrates, confusion or disorientation, blood urea nitrogen > 7 mmoL/L, leukocytes < 4000/mm^3^, platelets < 100,000 mm^3^, body temperature < 36 °C, hypotension requiring fluid repletion, metabolic acidosis, and high serum lactate level. Exclusion criteria were non-Caucasian ethnicity, pneumococcal pneumonia related to care or with an onset > 72 h after hospital admission, aspiration pneumonia in a comatose or trauma patient, and immunodeficiency (asplenia or splenectomy, chemotherapy, haematological malignancy within the past 6 months and not in complete remission, solid organ or bone marrow transplant, neutrophils < 1000/mm^3^ before the infection, HIV infection, Child C cirrhosis of the liver, or immunoglobulin deficiency).

### Date collection

For each patient, the study data were collected prospectively in an electronic case report form at admission and throughout the ICU stay. The following were recorded: demographics (age, gender, body mass index), comorbid conditions (McCabe score, Charlson comorbidity index), laboratory tests (white blood cells, platelets, lactate), severity of CAP (Fine score class, Sepsis-Related Organ Failure [SOFA] score, Simplified Acute Physiology Score version II [SAPS II], multilobar infiltrate), and management (catecholamines, need for renal replacement therapy, IMV, non-invasive ventilation, pre-hospital antibiotic therapy, time to antibiotic initiation, type of admission). The serotype and antibiotic susceptibility of recovered pathogens were also recorded. All patients were followed-up until death or hospital discharge. The cause of death was recorded.

Data on the initial empiric antibiotic therapy were collected, and the patients classified according to whether they received only a beta-lactam or also a macrolide or quinolone. Patients were considered to have received dual therapy if they had received it for at least 24 h. After recovery of the *S. pneumoniae* strain, the antibiotic therapy was adapted to the susceptibility test results. All patients had at least one paired blood culture at hospital admission before the introduction of antibiotic therapy, except in cases in which the treatment was started before hospitalisation. For the specific purposes of this post hoc study, we divided the patients into two groups depending on whether their initial blood cultures were positive (BC^+^ group) or negative (BC^−^ group) for *S. pneumoniae*.

### Serotypes and microbiology tests

Serotyping was performed at the French National Reference Centre for Pneumococci (FNRCP) using latex particles coated with pool, group, type, and factor antisera provided by the Statens Serum Institute (Copenhagen, Denmark). This panel of antisera enabled the recognition of 92 known serotypes. Pneumococcal strains with known serotypes from the Statens Serum Institute and the FNRCP collection were used as internal quality controls [20]. Potential differences in the distribution of serotypes between the BC^+^ and BC^−^ groups were assessed for patients with microbiologically documented pneumococcal CAP. Serotypes were then grouped according to their potential for causing invasive disease, as previously described [21–24], and their case fatality rate determined for each group, as previously reported [25, 26].

Antibiotic susceptibility testing was performed at the FNRCP. Susceptibility to penicillin G, amoxicillin, cefotaxime, and levofloxacin was determined using the agar dilution method and susceptibility to erythromycin using the disk diffusion method. In addition, the norfloxacin screen test was used according to the European Committee on Antimicrobial Susceptibility Testing (EUCAST) [27] to successfully distinguish wild-type pneumococcal strains from those with any acquired mechanism of resistance to fluoroquinolones [28]. The results were interpreted according to EUCAST breakpoints [27].

### Statistical analysis

The baseline characteristics of patients with blood cultures positive or negative for *S. pneumoniae* were compared using Fisher’s exact tests, Wilcoxon rank-sum tests, or Kruskal–Wallis tests. A potential difference in the distribution of serotypes according to the two groups, with or without positive blood cultures, was investigated.

The probability of hospital mortality was estimated using the cumulative incidence function estimator [29], with discharge alive as a competing risk, and cumulative incidences were compared using Gray’s test. Because this was an ancillary study, no prior statistical power calculation was performed. Given the sample size and prevalence of positive blood cultures, we computed that the study would have a power of 80% to detect an odds ratio of approximately 1.78, i.e. an increase in mortality from 15% for patients with a negative blood culture to 24% for patients with a positive blood culture.

The main analysis investigated potential differences in the association of baseline variables identified to be prognostic in the STREPTOGENE study and hospital mortality according to the blood culture results. The objective of this analysis was to highlight a potential interaction between one of the variables and mortality specific to the group of bacteraemic patients. We thus compared the odds ratios (ORs) obtained in a multivariable logistic regression model between the two groups based on blood culture, positive or not, by performing interaction tests. Models were generated from the multiply imputed dataset and pooled using Rubin’s rule. Finally, odds ratios for the association of blood culture positivity with hospital mortality were compared according to the first antibiotic treatment, both without adjustment and after adjustment for age, sex, BMI, and the McCabe and Charlson indices.

## Results

### Patients

Among the 614 patients included in the STREPTOGENE study between December 2008 and February 2012 in 51 French ICUs, all had at least one initial blood culture, of which 270 (44%) were positive for *S. pneumoniae*. In non-bacteraemic patients, S. pneumoniae was diagnosed by pneumococcal urinary antigen for 25% of cases. In almost all other cases, it was demonstrated from lung specimens**.** The baseline characteristics of the patients are presented in Table 1. The epidemiological characteristics were similar for the two groups. The patients were mostly male, with a median age > 60 years. The Fine score, median SOFA score, and median SAPS II indicated that most patients had severe acute illness, with a comparable distribution between the two groups. Leukopaenia was nearly twice as common in the group with positive blood cultures. Of note, more patients in the negative blood culture group had received pre-hospital antibiotics (27 [10] versus 56 [16.3], *p* = 0.024).

**Table 1:** Baseline characteristics of the 614 patients according to initial blood culture results

Variable	Positive blood culture (*N* = 270)	Negative blood culture (*N* = 344)	*p* value
Age, years, median (range)	62 (19 to 96)	64 (21 to 99)	0.080
Gender, *n* (%)			0.80
Female	106 (39.3)	131 (38.1)	
Male	164 (60.7)	213 (61.9)	
BMI, kg/m^2^, median (*Q*_1_ to *Q*_3_)	24.2 (21.2 to 27.6)	24.5 (21.3 to 29.0)	0.37
*N* missing	25	23	
Admission, *n* (%)			0.35
Direct admission to ICU	28 (10.4)	46 (13.4)	
Transfer from ER	202 (74.8)	257 (74.7)	
Transfer from another ward	40 (14.8)	41 (11.9)	
McCabe score, *n* (%)			0.27
1	243 (90.0)	319 (92.7)	
2	26 (9.6)	25 (7.3)	
3	1 (0.4)	0 (0)	
Pneumonia severity index, *n* (%)			0.033
II	10 (3.7)	27 (7.8)	
III	35 (13.0)	28 (8.1)	
IV	89 (33.0)	102 (29.7)	
V	136 (50.4)	187 (54.4)	
SOFA score, median (*Q*_1_ to *Q*_3_)	7 (4 to 11)	7 (4 to 10)	0.14
SAPS II, median (*Q*_1_ to *Q*_3_)	45 (32 to 58)	43 (33 to 56)	0.65
Charlson comorbidity index, *n* (%)			0.20
0–1	51 (18.9)	50 (14.5)	
2	47 (17.4)	47 (13.7)	
3	44 (16.3)	62 (18.0)	
≥ 4	128 (47.4)	185 (53.8)	
WBC < 4 × 10^9^/L, *n* (%)	69 (25.9)	47 (13.7)	0.0002
*N* missing	4	2	
Platelets ≤ 100 × 10^9^/L, *n* (%)	48 (18.0)	42 (12.5)	0.065
*N* missing	4	7	
Lactate (mmol/L), *n* (%)			0.11
< 2	67 (27.1)	114 (35.2)	
2–4	117 (47.4)	132 (40.7)	
> 4	63 (25.5)	78 (24.1)	
*N* missing	23	20	
Catecholamines, *n* (%)	124 (45.9)	154 (44.8)	0.81
Need for RRT, *n* (%)	14 (5.2)	8 (2.3)	0.079
Invasive mechanical ventilation, *n* (%)	136 (50.4)	171 (49.7)	0.94
Non-invasive ventilation, *n* (%)	69 (25.6)	102 (29.7)	0.28
Pulmonary infection, *n* (%)			> 0.99
1 lobe	95 (35.2)	121 (35.2)	
2 lobes	69 (25.6)	88 (25.6)	
Bilateral	106 (39.3)	135 (39.2)	
Pre-hospital antibiotic therapy, *n* (%)	27 (10.0)	56 (16.3)	0.024
Time to antibiotics, *n* (%)			0.99
< 3 h	112 (48.1)	140 (47.9)	
3–6 h	60 (25.8)	77 (26.4)	
> 6 h	61 (26.2)	75 (25.7)	
*N* missing	37	52	

*BMI* body mass index, *ICU* intensive care unit, *ER* emergency room, *SOFA* Sequential Organ Failure Assessment, *SAPS II* Simplified Acute Physiology Score II, *WBC* white blood cells, *RRT* renal replacement therapy

### Serotypes and antibiotic susceptibility

There was a significant difference in the serotype distribution between the two groups (adjusted *p* = 0.003) (Table 2). Serotypes 7F, 1, and 12F were more common in the BC^+^ than BC^−^ group (18% vs. 11%, 9% vs. 1.6%, and 9% vs. 2.4%, respectively). Serotypes 3, 7F, and 19A were the most common in both groups. Invasive serotypes were significantly more common in the BC^+^ group (adjusted *p* = 0.002). On the other hand, serotypes associated with high case fatality rates were more common in the BC^−^ group (adjusted *p* < 0.0001).

**Table 2:** Serotype distribution and antibiotic susceptibility according to initial blood culture results

Variable	Positive blood culture (*N* = 212)	Negative blood culture (*N* = 127)	*p* value	Adjusted *p* value^a^
Serotype, *n* (%)			< 0.0001	0.003
3	45 (21.2)	36 (28.3)		
7F	38 (17.9)	14 (11.0)		
19A	31 (14.6)	18 (14.2)		
12F	19 (9.0)	3 (2.4)		
1	19 (9.0)	2 (1.6)		
6C	6 (2.8)	4 (3.1)		
11A	1 (0.5)	9 (7.1)		
Other	53 (25.0)	41 (32.3)		
Serotype invasiveness^b^, *n* (%)			0.002	0.002
High	166 (78.3)	78 (61.4)		
Low	13 (6.1)	20 (15.7)		
Undetermined	33 (15.6)	29 (22.8)		
Serotype case fatality rate^c^, *n* (%)			< 0.0001	< 0.0001
Low	72 (34.0)	19 (15.0)		
Intermediate	30 (14.2)	11 (8.7)		
High	102 (48.1)	86 (67.7)		
Other	8 (3.8)	11 (8.7)		
Penicillin susceptible, *n* (%)	168 (79.2)	92 (72.4)	0.18	0.14
Amoxicillin susceptible, *n* (%)	198 (93.4)	107 (84.3)	0.009	0.010
Cefotaxime susceptible, *n* (%)	206 (97.2)	123 (96.9)	> 0.99	0.70
Erythromycin susceptible, *n* (%)	163 (76.9)	93 (73.2)	0.51	0.41
Levofloxacin susceptible, *n* (%)	210 (99.1)	127 (100.0)	0.53	–

Data were available for 339 patients

^a^Adjusted for age category, sex, BMI category, and McCabe and Charlson scores

^b^Serotype invasiveness: high (OR > 1, *p* < 0.05), serotypes 1, 3, 4, 5, 7F, 12F, 14, 18C, 19A, and 9L; low (OR < 1, *p* < 0.05), serotypes 6A, 6C, 10A, 11A, 15A, 15C, 23B, 24F, and 37; and undetermined (OR < 1 or OR > 1 with *p* > 0.05), serotypes 8, 9A, 9N, 9V, 16F, 17F, 18A, 19F, 20, 22F, 23F, 29, 31, 33F, 34, 35B, and 35F

^c^Serotype case fatality rate: low (serotypes 1, 4, 5, 7F, and 8), intermediate (serotypes 9V, 12F, 14, and 22F), or high (serotypes 3, 6A, 6B, 6C, 9N, 11A, 19A, 19F, and 23F)

Amoxicillin-susceptible *S. pneumoniae* strains were more common in the BC^+^ group (93.4% vs. 84.3%, adjusted *p* = 0.010). The susceptibility patterns for penicillin, cefotaxime, erythromycin, and levofloxacin were similar in both groups.

### Antibiotic treatment

Probabilistic antibiotic therapy was appropriate for all included patients. Most patients (70%) received dual antibiotic therapy with a beta-lactam and a fluoroquinolone or macrolide. Fewer than a quarter of the patients received a beta-lactam alone. The distribution of the various therapeutic modalities was comparable for the two groups (*p* = 0.54). When used for dual therapy, macrolides and fluoroquinolones were evenly distributed between the two groups, with approximately 35% of patients for each (Additional file 1).

### Mortality

**Fig. 1:**
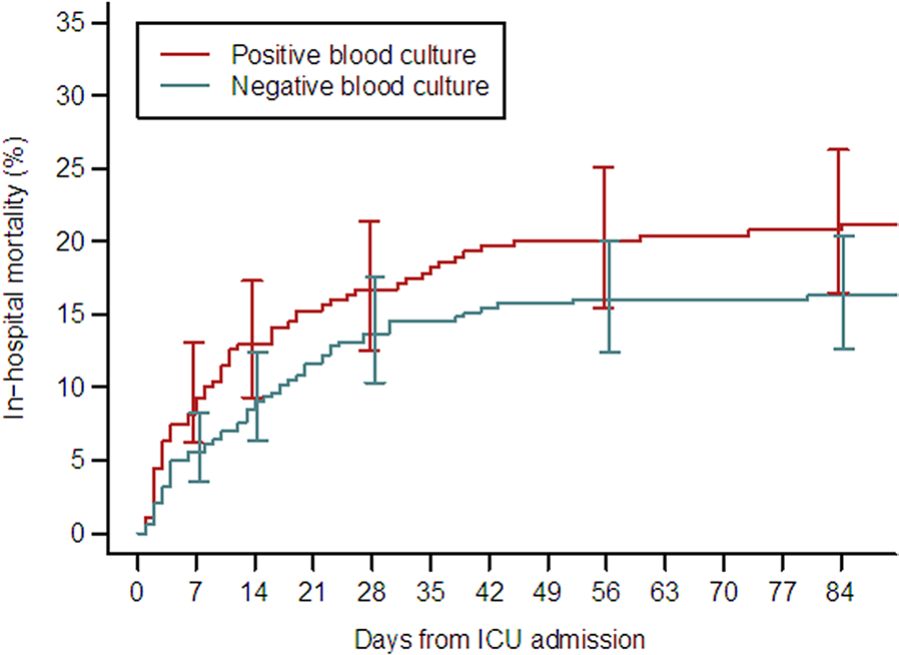
Probability of hospital mortality according to blood culture result (*p* = 0.11)

There was no significant difference in hospital mortality between the two groups (Fig. 1), nor in early mortality on day 5 (7.4% versus 4.9%) (Table 3). The results for the primary endpoint are presented in Table 4. Comparative analysis of the mortality odds ratios for the different variables according to blood culture at admission did not show any significant differences. Thus, none of these variables showed a stronger prognostic link for bacteraemic than non-bacteraemic patients. In particular, the association between mortality and time to antibiotic therapy (pre-hospital antibiotic therapy and time to antibiotics > 6 h) was not significantly different for the two groups. Furthermore, mortality was not higher in patients > 65 years of age in the BC^+^ group. The causes of death were similar for both groups (Table 3), with multi-organ failure as the leading cause of death (52%).

**Table 3:** Mortality and causes of death

Variable	Positive blood culture (*N* = 270)	Negative blood culture (*N* = 344)
Day-5 mortality, *n* (%)	20 (7.4)	17 (4.9)
Hospital mortality, *n* (%)	58 (21.5)	58 (16.9)
Cause of death, *n* (%)		
Multi-organ failure	30 (51.7)	23 (39.7)
Multi-organ failure + hypoxaemia	3 (5.2)	6 (10.3)
Hypoxaemia	5 (8.6)	7 (12.1)
Shock	5 (8.6)	3 (5.2)
Neurological	4 (6.9)	3 (5.2)
Cardiac	1 (1.7)	2 (3.4)
Severe digestive disorder	2 (3.4)	1 (1.7)
Other pulmonary	1 (1.7)	1 (1.7)
Acute respiratory distress syndrome	1 (1.7)	1 (1.7)
WLST (unknown cause)	6 (10.3)	9 (15.5)
Unknown	0 (0)	2 (3.4)

*WLST* withdrawing life-sustaining therapy

**Table 4:** Associations of baseline variables with hospital deaths in the groups with and without bacteraemia

Variable	Positive blood culture (*N* = 270)	Negative blood culture (*N* = 344)	*p* value (interaction)
Age (years)			
18–50	1	1	–
51–65	1.70 (0.56 to 5.12)	0.77 (0.21 to 2.76)	0.36
> 65	4.32 (1.47 to 12.7)	2.33 (0.70 to 7.77)	0.45
Male gender	1.70 (0.79 to 3.67)	1.84 (0.85 to 3.98)	0.89
McCabe score ≥ 2	1.23 (0.42 to 3.61)	4.03 (1.24 to 13.2)	0.14
SAPS II (per unit)	1.04 (1.01 to 1.07)	1.04 (1.02 to 1.07)	0.79
Platelets ≤ 100 × 10^9^/L	1.59 (0.62 to 4.10)	2.66 (1.02 to 6.92)	0.24
Lactates (mmol/L)			
< 2	1	1	–
2–4	0.76 (0.28 to 2.04)	1.77 (0.65 to 4.84)	0.18
> 4	1.50 (0.52 to 4.34)	4.29 (1.45 to 12.7)	0.46
Bilateral pulmonary infection	1.63 (0.77 to 3.43)	1.90 (0.93 to 3.88)	0.77
Shock	2.19 (0.86 to 5.59)	1.95 (0.86 to 4.40)	0.85
Invasive mechanical ventilation	1.11 (0.38 to 3.21)	3.06 (1.24 to 7.59)	0.15
Pre-hospital antibiotic therapy	1.20 (0.35 to 4.06)	1.09 (0.41 to 2.91)	0.91
Time to antibiotics (h)			
< 3	1	1	–
3–6	0.56 (0.19 to 1.59)	0.73 (0.27 to 2.02)	0.71
> 6	1.23 (0.50 to 3.02)	1.66 (0.71 to 3.90)	0.63

*SAPS II* Simplified Acute Physiology Score II

**Fig. 2:**
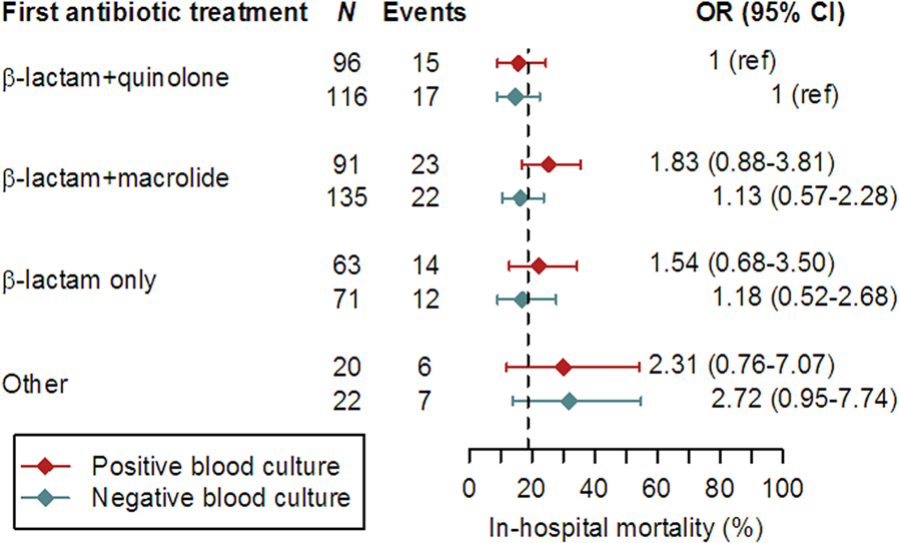
Hospital mortality according to blood culture result and first antibiotic treatment

The dashed lines in Fig. 2 show the overall hospital mortality rate. ORs are given for each treatment relative to beta-lactam plus fluoroquinolone for each of the two groups. Finally, there was no evidence of an interaction between blood culture group and type of antibiotic treatment on hospital mortality (*p* = 0.74). Indeed, although mortality appeared to higher in the “other treatments” category (*p* = 0.12) and the BC^+^ group (*p* = 0.15), the differences were not statistically significant. Adjustment for age, sex, BMI, McCabe score, and Charlson comorbidity index did not alter these results (*p* = 0.66, *p* = 0.13, and *p* = 0.087, respectively).

## Discussion

In this post hoc study of data from STREPTOGENE study, blood culture positivity was not associated with mortality in a large prospective cohort of immunocompetent Caucasian patients admitted to the ICU for pneumococcal CAP. Furthermore, the associations linking known prognostic parameters to mortality were similar in the BC^+^ and BC^−^ groups. The differences in serotype found between the two groups suggest that specific serotypes may be more frequently associated with bacteraemia. Antibiotic regimens were similar for the two groups and none of the antibiotic regimens was associated with lower mortality in either.

At admission, 44% of the STREPTOGENE population had bacteraemia, consistent with the results of earlier studies with patients in and outside of the ICU [3, 4, 30, 31]. Two ICU studies also found no association between bacteraemia and mortality [3, 4]. Thus, a large retrospective study of two prospectively-acquired databases showed similar organ failure features and hospital mortality rates for patients with and without bacteraemia [3]. These results challenge the earlier belief that bacteraemia carries a poor prognosis [5–9]. Bacteraemia was thus included in the PIRO score developed to assess the severity of CAP in the ICU [32]. Our findings and those of other recent studies [3, 10, 11] suggest that the PIRO score may not be suitable for the specific population of ICU patients with pneumococcal CAP. It is important that bacteraemia no longer be viewed as contributing to an adverse prognosis in this population.

The serotype distribution showed a number of interesting features. Serotypes generally considered to be responsible for invasive pneumococcal disease were more common in the BC^+^ group, as expected, since bacteraemia is among the manifestations of invasiveness. Discrepancies between serotype invasiveness and mortality have been previously reported [25, 33, 34] and remain a pathophysiological mystery. However, on the contrary, serotypes generally reported to be associated with high fatality rates were more common in the BC^−^ group. This finding supports the absence of an association between bacteraemia and mortality. Amoxicillin-susceptible isolates were more common in the BC^+^ group. However, one study has suggested that the metabolic requirements of developing resistance to antibiotics may result in the loss of invasive potential [35].

In the early 2000s, three observational studies [13–15] launched the debate on the possible superiority of dual therapy with a beta-lactam and macrolide for the treatment of pneumococcal infections with bacteraemia. Macrolides may be beneficial due to their immunomodulatory activity on the proinflammatory responses of leukocytes and other host cells [36, 37]. This effect was demonstrated in vitro, but most in vivo studies involved patients with chronic airway inflammation and not those with CAP [38, 39]. Subsequently, no prospective randomised study confirmed the superiority of the beta-lactam-macrolide combination in ICU patients with pneumococcal CAP and bacteraemia. Most studies focused on whether dual therapy was beneficial when given as the probabilistic treatment of CAP that required admission to the ICU or a medical ward [12, 40–42]. In our prospective observational study of immunocompetent patients, mortality was not significantly different between monotherapy and dual therapy in either of the blood culture groups. Importantly, the antibiotic regimens were similar for the BC^+^ and BC^−^ groups.

Our study had several limitations. The observational prospective design allowed us to describe epidemiological and prognostic features, as well as serotypes, in a large cohort. One of the important limitations concerns the significant proportion of patients who received pre-hospital antibiotic therapy, which may have influenced the positivity of blood cultures on admission, leading to a misclassification bias. However, the absence of randomisation precludes the drawing of conclusions about the relative efficacy of the various antibiotic regimens. Conceivably, dual therapy was given more often to patients with markers of severe disease. We did not have accurate data on the duration of antibiotic therapy. Although our study included 614 patients, only 349 isolates were serotyped, potentially limiting our ability to detect associations between serotypes and bacteraemia. Concerning the serotypes, the influence of pneumococcal vaccination on their incidence was not studied because it was not possible to retrospectively know the vaccination status of the patients. The database was compiled during the main study, between 2008 and 2012. Although there has not been a therapeutic revolution in the treatment of pulmonary infections in the ICU, the conclusions need to be interpreted in light of the fact that practices have changed in the 10 years since the constitution of this cohort. Finally, the use of a previously collected cohort did not allow us to obtain all of the variables that we would have liked, such as the use of corticosteroids, mortality after hospital discharge, occurrence of meningitis or endocarditis, days of mechanical ventilation, length of stay, superinfection, and complications during the hospital stay.

## Conclusion

This study did not show any specific prognostic factors in the event of bacteraemia in a population of immunocompetent Caucasian adults managed in the ICU for severe pneumococcal CAP. The association of predefined prognostic factors with mortality was similar for the group with and that without bacteraemia. Patients with bacteraemia more often had invasive serotypes, as expected, but less often had serotypes associated with high case fatality rates. Keeping in mind the limitations of this study, we found no evidence that dual antibiotic therapy is superior to monotherapy in severe bacteraemic pneumococcal CAP. Bacteraemia is not a factor related to mortality and there is no reason to change the therapeutic management of patients. However, a randomised study is needed to definitively address this question.

## Supplementary Information

**Additional file 1: Figure S1.** First antibiotic treatment prescribed to patients with and without bacteraemia. Details of probabilistic antibiotic therapy according to the status of blood cultures.

## Data Availability

The datasets used and/or analysed during the current study are available from the corresponding author on reasonable request.
