# Supplementary figures and images for: Comparison of prognostic factors between bacteraemic and non-bacteraemic critically ill immunocompetent patients in community-acquired severe pneumococcal pneumonia: a STREPTOGENE sub-study
Source: Ann Intensive Care. 2021 Oct 24;11:148. doi: 10.1186/s13613-021-00936-z (PMC8542522; doi:10.1186/s13613-021-00936-z)

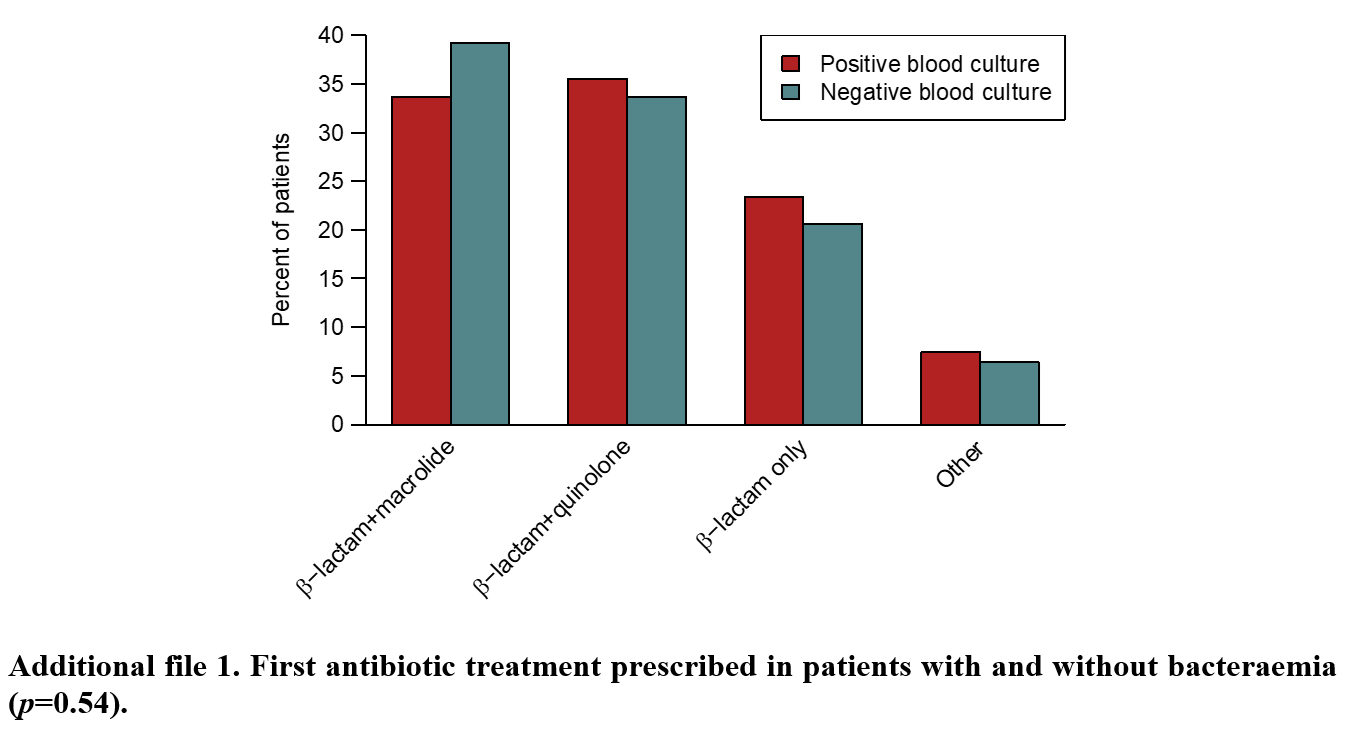

Supplement: Supplementary file 1 — Additional file 1: Figure S1. First antibiotic treatment prescribed to patients with and without bacteraemia. Details of probabilistic antibiotic therapy according to the status of blood cultures. [file 13613_2021_936_MOESM1_ESM.tif]
